# The SWI/SNF chromatin-remodeling factors BAF60a, b, and c in nutrient signaling and metabolic control

**DOI:** 10.1007/s13238-017-0442-2

**Published:** 2017-07-07

**Authors:** Ruo-Ran Wang, Ran Pan, Wenjing Zhang, Junfen Fu, Jiandie D. Lin, Zhuo-Xian Meng

**Affiliations:** 1 Department of Pathology and Pathophysiology, Key Laboratory of Disease Proteomics of Zhejiang Province, School of Medicine, Chronic Disease Research Institute of School of Public Health, Zhejiang University, Hangzhou, 310058 China; 2 Children’s Hospital, Zhejiang University School of Medicine, Hangzhou, 310003 China; 3 Life Sciences Institute and Department of Cell & Developmental Biology, University of Michigan Medical Center, Ann Arbor, MI 48109 USA

**Keywords:** BAF60a, BAF60b, BAF60c, chromatin-remodeling, SWI/SNF, energy metabolism, nutrient sensing, glucose, lipid, skeletal muscle, liver

## Abstract

Metabolic syndrome has become a global epidemic that adversely affects human health. Both genetic and environmental factors contribute to the pathogenesis of metabolic disorders; however, the mechanisms that integrate these cues to regulate metabolic physiology and the development of metabolic disorders remain incompletely defined. Emerging evidence suggests that SWI/SNF chromatin-remodeling complexes are critical for directing metabolic reprogramming and adaptation in response to nutritional and other physiological signals. The ATP-dependent SWI/SNF chromatin-remodeling complexes comprise up to 11 subunits, among which the BAF60 subunit serves as a key link between the core complexes and specific transcriptional factors. The BAF60 subunit has three members, BAF60a, b, and c. The distinct tissue distribution patterns and regulatory mechanisms of BAF60 proteins confer each isoform with specialized functions in different metabolic cell types. In this review, we summarize the emerging roles and mechanisms of BAF60 proteins in the regulation of nutrient sensing and energy metabolism under physiological and disease conditions.

## INTRODUCTION

Metabolic syndrome has become a global epidemic that markedly increases the risk for type 2 diabetes, cardiovascular diseases, and non-alcoholic fatty liver disease (NAFLD). Both genetic and environmental factors contribute to the pathogenesis of metabolic syndrome. In mammalian cells, the approximately 1.7 meters of genomic DNA are compacted more than 5,000 fold in order to squeeze into the nucleus, which is very important for the stability of chromatin structure. Chromatin is a complex of macromolecules found in eukaryotic cells. The basic structural unit of chromatin is nucleosome consisting of a segment of DNA wound in sequence around eight histone protein cores. Under certain circumstances such as DNA replication and transcription, the highly compacted chromatin structures have to be dissolved to allow the recruitment of replication or transcription complexes to local chromatin regions. At least three major epigenetic mechanisms regulate the landscape of chromatin and the accessibility of DNA to transcription factors and other proteins: DNA methylation, chromatin remodeling, and histone modification (Holliday, 2006; Cairns, 2009). Recent studies suggest that epigenetic processes play an important role in metabolic adaptation and the development of metabolic disorders through integrating the environmental signals (such as nutrient, hormone, exercise, and inflammation) to orchestrate the transcriptional networks involved in metabolic regulation (Keating and El-Osta, 2015).

The ATP-dependent chromatin-remodeling complexes can be divided into five main families: ISWI (imitation switch), NuRD/Mi2/CHD (chromodomain helicase DNA-binding), INO80, SWR1, and SWI/SNF (switching defective/sucrose nonfermenting); the latter is the focus of this review. Recently, we and other groups have provided strong evidence suggesting that the SWI/SNF chromatin-remodeling factors, BAF60a, b, and c in particular, serve as key regulators in cell nutrient sensing, metabolic reprogramming, and the control of metabolic homeostasis in physiological and disease conditions (Lee et al., 2007; Li et al., 2008; Tao et al., 2011; Cotton et al., 2012; Meng et al., 2013; Wang et al., 2013; Meng et al., 2014; Meng et al., 2015; Lal et al., 2016; Meng et al., 2017). In this review, we focus on the roles and mechanisms of BAF60 proteins in the regulation of energy homeostasis and the development of metabolic disorders.

## THE SWI/SNF COMPLEX

SWI/SNF was first discovered as a large multi-subunit complex in yeast, and named for switching defective/sucrose nonfermenting (Neigeborn and Carlson, 1984; Peterson and Herskowitz, 1992). In 1994, Brahma (Brm), the homolog of yeast transcriptional activator SWI2/SNF2, was identified in Drosophila (Elfring et al., 1994). In 1996, Wang et al. demonstrated that SWI/SNF complexes are present in multiple forms made up by 9–12 proteins in mammals (Wang et al., 1996). Since then, at least 11 subunits of SWI/SNF complex have been discovered, including 2 enzymatic ATPase subunits (Brm and Brg1) and 10 Brg1/Brm associated factors (BAFs), which are named according to their molecular weights (Sudarsanam and Winston, 2000; Wu et al., 2009). These SWI/SNF subunits, encoded by highly conserved genes, can be assembled into distinct chromatin-remodeling complexes in different cell types and developmental stages, thereby playing an essential role in tissue development (Ho and Crabtree, 2010). In addition, recent studies demonstrated that genes encoded for the SWI/SNF chromatin-remodeling complex are mutated in approximately 20% of all human tumors (Kadoch et al., 2013), and are considered to be critical epigenetic regulators of tumorigenesis due to their pleiotropic roles in the regulation of metabolism, cell cycle, and oncogenic pathway (Wiegand et al., 2010; Li et al., 2011; Varela et al., 2011; Wilson and Roberts, 2011; Shain et al., 2012; Shain and Pollack, 2013; Masliah-Planchon et al., 2015).

Mammalian SWI/SNF complexes are composed of one of two catalytic ATPase subunits (Brg1 or Brm) and 4–12 additional subunits known as BAFs. While BAF47, BAF170, and BAF155 form the core complex with Brg1 or Brm, other BAFs confer an extensive diversity of the complexes with specialized functions in different tissues through polymorphic assembly. During the past decade, most studies have focused on subunits in the core complex, revealing critical roles of the SWI/SNF in tissue development, stem cell renewal, and cancer pathogenesis. However, the physiological functions of other tissue specific subunits, which are responsible for the functional diversity of different SWI/SNF complexes, remain largely unknown (Hargreaves and Crabtree, 2011).

## BAF60A, B, and C

Among all the subunits, the BAF60 subunit is unique and important since it serves as a linker between the SWI/SNF core complex and transcription factors to regulate target gene expression (Surabhi et al., 1999; Hsiao et al., 2003; Debril et al., 2004; Flajollet et al., 2007; Takeuchi et al., 2007; Lamba et al., 2008; Oh et al., 2008). BAF60 subunit has three members, BAF60a, b, and c. The distinct tissue distribution patterns and regulatory mechanisms of BAF60s grant each isoform with specialized functions. We and other research groups have recently demonstrated that BAF60s display distinct expression profiles in metabolic tissues, and are exclusively required for the regulation of energy metabolism in physiological and disease states in a tissue specific manner. Here we summarize our current understanding of the important roles and underlying mechanisms of BAF60s in metabolic regulation and the pathogenesis of metabolic diseases.

## BAF60A IN METABOLIC CONTROL AND DISEASES

**Figure 1:**
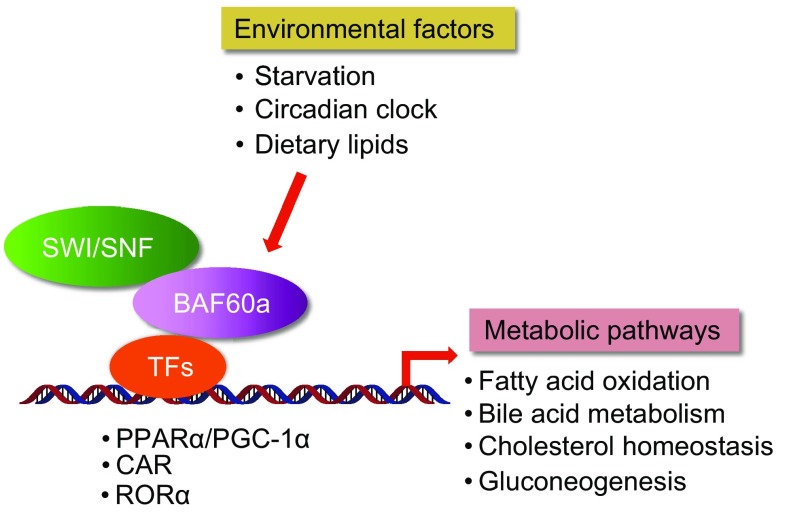
**Metabolic regulation by BAF60a in liver**. Hepatic BAF60a senses and integrates the environmental factors, such as starvation, circadian clock, and dietary fat consumption, to transcriptional reprogramming and metabolic adaptations, through its direct interaction with specific transcriptional factors/cofactors and SWI/SNF-mediated chromatin remodeling

BAF60a, encoded by *Smarcd1* gene, is highly expressed in liver, brain, and adipose tissues. Our previous studies demonstrated that BAF60a regulates hepatic fatty acid β-oxidation (FAO) through its interaction with PPARα and PPARγ coactivator-1α (PGC-1α) (Li et al., 2008) (Fig. 1). In addition, Tao et al. found that BAF60a is rhythmically expressed in mouse liver, and plays an important role in integrating peripheral circadian clock and energy metabolism in mammals (Tao et al., 2011) (Fig. 1). In a recent study, we demonstrated that hepatic BAF60a serves as a key component of a dietary sensitive pathway that conveys nutritional signals to transcriptional reprogramming to maintain whole body cholesterol homeostasis (Meng et al., 2015).

### BAF60a and fatty liver disease

As one of the major metabolic organs, liver plays a key role in the control of systemic glucose and lipid homeostasis (Hardie and Ashford, 2014; Zhang et al., 2016). Liver regulates several major aspects of lipid metabolism, including *de novo* lipogenesis (DNL), FAO, as well as lipoprotein synthesis, uptake, and secretion. Dysregulation of hepatic lipid metabolism leads to NAFLD, which is a common and severe complication of obesity and type 2 diabetes (Kotronen and Yki-Jarvinen, 2008). NAFLD is the most prevalent cause of chronic liver injury and featured by an ectopic accumulation of triglycerides (TG), which comprises a disease spectrum ranging from simple fatty liver to non-alcoholic steatohepatitis (NASH), advanced fibrosis and cirrhosis. A better understanding of the molecular mechanisms mediating the regulation of lipid metabolism in the liver is critical for the design of new therapeutic interventions for NAFLD.

PGC-1α is an inducible coactivator for nuclear hormone receptors and other transcriptional factors that regulates several major metabolic pathways including mitochondrial oxidative phosphorylation (OXPHOS) (Wu et al., 1999; Lehman et al., 2000) and hepatic gluconeogenesis (Herzig et al., 2001; Yoon et al., 2001). Through a genome-wide high-throughput coactivation screen, Li et al. identified BAF60a as an interaction partner of PGC-1α in the liver (Li et al., 2008). This study revealed that BAF60a is a key factor that bridges the SWI/SNF chromatin-remodeling complex and the fatty acid β-oxidation gene program in the liver. PGC-1α mediates the recruitment of BAF60a to PPARα binding sites, leading to transcriptional induction of genes involved in FAO. Adenoviral-mediated expression of BAF60a dose-dependently stimulates the transcription of genes involved in peroxisomal and mitochondrial fatty acid β-oxidation. Further *in vivo* studies demonstrated that BAF60a gain-of-function promotes FAO and ameliorates liver steatosis in diet-induced and genetically obese mice. In contrast, RNAi-mediated knockdown of BAF60a in the liver attenuates the induction of genes involved in FAO in response to starvation, leading to TG accumulation and liver steatosis. These results define BAF60a as a key regulator of hepatic FAO function and liver steatosis.

### BAF60a couples circadian rhythms and energy metabolism

In mammals, many aspects of energy metabolism including glucose and lipid metabolism are coordinated by biological clocks residing in both brain and the peripheral tissues (Rutter et al., 2002; Wijnen and Young, 2006). By coordinating the metabolic and circadian transcriptional programs, PGC-1α serves as a molecular switch that synchronizes metabolic functions to the circadian clocks in mouse liver (Liu et al., 2007). As mentioned above, BAF60a is an interaction partner for PGC-1α in the regulation of hepatic lipid metabolism (Li et al., 2008). More interestingly, BAF60a is also rhythmically expressed in mouse liver, raising the possibility that BAF60a may also play a critical role in integrating the circadian signals and metabolic programs in the liver. Consistent with this hypothesis, Tao et al. demonstrated that adenoviral-mediated knockdown of BAF60a in liver significantly altered the rhythmic expression of clock genes including *Bmal1*, *Period 1* (*Per1*), *Per2*, *Rev-erbα*, and *Cryptochrome 1* (*Cry1*), and the genes involved in some major metabolic pathways such as gluconeogenesis, FAO, and mitochondrial respiration (Tao et al., 2011). Further mechanistic studies revealed that BAF60a induces *Bmal1* and *G6Pase* transcription by forming a transcriptional complex with retinoid-related orphan receptor alpha (RORα), allowing the recruitment of the SWI/SNF complex to activate local chromatin. These results suggest that BAF60a plays an important role in coupling circadian cues to metabolic gene expression programs and functions.

MiR-122, a hepatocyte-enriched microRNA previously linked to the regulation of cholesterol and lipid metabolism, also exhibits rhythmic expression pattern. It has been reported that depletion of miR-122 in the liver disrupts the mRNA expression of genes associated with cholesterol and lipid metabolism. Interestingly, PPARβ/δ and BAF60a were recently identified as miR-122 targets (Gatfield et al., 2009), suggesting an involvement of PPARβ/δ and BAF60a in coupling the circadian clocks to miR-122-mediated metabolic regulation.

### BAF60a and whole body cholesterol homeostasis

Atherosclerosis is responsible for most of the cardiovascular mortality and morbidity in Western societies (Ross, 1993). Elevated plasma level of low-density lipoprotein (LDL)-cholesterol is an independent risk factor for atherosclerosis (Glass and Witztum, 2001; Steinberg, 2002). However, the currently available cholesterol lowering drugs such as statins and bile acid sequestrants could not reduce plasma cholesterol concentrations to the levels recommended by clinical guidelines in a large proportion of patients (Grundy et al., 2004; Waters et al., 2009), suggesting that other mechanisms may also play significant roles in the regulation and maintenance of cholesterol homeostasis.

Liver plays a central role in the control of systemic cholesterol homeostasis by regulating cholesterol biosynthesis, uptake and secretion in lipoproteins, and its catabolism and excretion as bile acids (Chiang, 2009; Goldstein and Brown, 2015). However, how the dietary signals are sensed and integrated into transcriptional reprogramming and metabolic functions to maintain systemic cholesterol homeostasis remains largely unknown. Recent ChIP-Seq studies revealed that, among the candidate genes identified by GWAS as potential contributors of dyslipidemia (Kathiresan et al., 2009), over 70% (32 out of 45) are bound by components of the SWI/SNF complex within 2 kb of the respective transcriptional start sites (Euskirchen et al., 2011; Dunham et al., 2012), suggesting that SWI/SNF chromatin remodeling factors may serve as core components of dietary sensing machinery that couples nutrient signals to metabolic reprogramming involved in lipid metabolism. To test this possibility, we analyzed the expression of SWI/SNF subunits in the liver from mice fed standard chow or western diet (WD), the latter contains a high amount of cholesterol and has been widely used to induce hypercholesterolemia. While the expression of core SWI/SNF subunits, including Brg1, BAF170, BAF155, and BAF53a, and the other two BAF60 proteins BAF60b and BAF60c remained similar between the two groups, mRNA and protein levels of BAF60a were significantly elevated following WD feeding (Meng et al., 2015). These data suggest that BAF60a may serve as a diet-sensitive component of the SWI/SNF complexes in the liver that links dietary cholesterol intake to whole body cholesterol homeostasis. Consistently, hepatocyte-specific deficiency of BAF60a protected the mice from diet-induced hypercholesterolemia and atherosclerosis, accompanied by reduced liver bile acid production and lower intestinal cholesterol absorption. Conversely, adenoviral-mediated overexpression of BAF60a elevates plasma cholesterol levels. Further gene expression analyses revealed that BAF60a induces the expression of CAR, which regulates bile acid detoxification by stimulating the expression of hepatic genes responsible for the modification, conjugation, and transportation of bile acids (Pascussi et al., 2008; Li and Chiang, 2013). Mechanistically, BAF60a physically interacts with CAR and forms a feedforward regulatory loop to stimulate the expression of CAR itself and its downstream target genes involved in bile acid metabolism. BAF60a is required for the recruitment of the SWI/SNF chromatin-remodeling complexes to the proximal promoter regions of CAR and its target genes to facilitate an epigenetic transition from repressive to active states. These studies define BAF60a as the central node of a dietary sensitive regulatory pathway that transmits nutritional signals to the gene expression program that governs bile acid metabolism and cholesterol homeostasis. Therapeutic intervention of this pathway may be used as a new way to treat diet-induced hypercholesterolemia and reduce the risk of atherosclerosis.

## BAF60C IN METABOLIC CONTROL AND DISEASES

**Figure 2:**
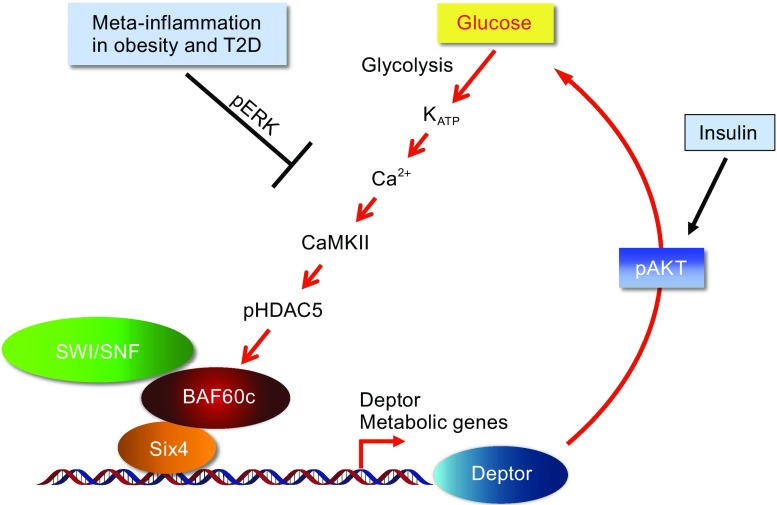
**Metabolic regulation by BAF60c in skeletal muscle**. BAF60c drives glycolytic muscle metabolism through Deptor-mediated AKT activation. Mechanistically, BAF60c forms a transcriptional complex with Six4 and recruits the SWI/SNF complex to the proximal Deptor promoter to induce Deptor gene expression. This pathway is important for muscle glucose metabolism and whole body glucose homeostasis in both physiological and diabetic states. In severe diabetic condition, expression of BAF60c and Deptor is downregulated due to activation of ERK pathway by meta-inflammation. Rescue of this pathway by either treatment with an ERK inhibitor or transgenic expression of BAF60c in skeletal muscle ameliorates insulin resistance and improves whole body glucose homeostasis in HFD-induced or genetically obese mice. Under physiological condition, BAF60c acts as a glucose sensor in skeletal muscle. Glucose triggers K_ATP_ channel-dependent Ca^2+^ response, which elicits the phosphorylation and nuclear exclusion of HDAC5 downstream of CaMKII activation, resulting in BAF60c and Deptor induction and insulin-independent AKT activation. This glucose sensing pathway works in concert with insulin signaling pathway to maintain postprandial glucose homeostasis

**Figure 3:**
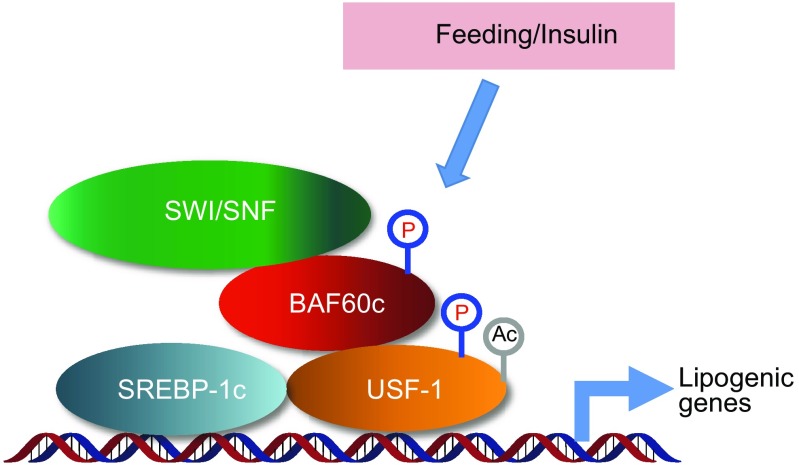
**Regulation of insulin-induced lipogenesis by BAF60c in mouse liver**. Upon insulin treatment, BAF60c is phosphorylated and translocated to the nucleus, which leads to its direct interaction with phosphorylated/ acetylated USF-1, allowing the recruitment of SWI/SNF chromatin-remodeling complex and transcriptional induction of lipogenic genes

BAF60c, encoded by *Smarcd3* gene, is preferentially expressed in skeletal muscle, brain, and heart, and has been identified as an important regulator of heart and retina development (Lickert et al., 2004; Lamba et al., 2008). Our recent studies demonstrated that BAF60c is the core component of a novel regulatory pathway that drives glycolytic metabolism in skeletal muscle (Meng et al., 2013) (Fig. 2). Expression of BAF60c and its downstream target gene Deptor is downregulated in skeletal muscle from diabetic mice, linking obesity associated-inflammation to skeletal muscle metabolism and systemic glucose homeostasis (Meng et al., 2014) (Fig. 2). Transgenic rescue of BAF60c expression in skeletal muscle restores Deptor-mediated AKT activation and improves whole body glucose homeostasis in diet-induced and genetically obese mice. Furthermore, in a recent study, we discovered BAF60c-Deptor-AKT as the target of a glucose sensing pathway in skeletal muscle that acts in concert with insulin signaling pathway to maintain postprandial glucose homeostasis (Meng et al., 2017) (Fig. 2). In addition, it has also been reported that BAF60c plays an important role in insulin-induced lipogenesis in mouse liver (Wang et al., 2013) (Fig. 3).

### BAF60c and glycolytic muscle metabolism

As one of the largest organs in normal human body, skeletal muscle is the major site of postprandial glucose disposal (Thiebaud et al., 1982; Ferrannini et al., 1988). As such, impaired skeletal muscle insulin action and glucose utilization are the primary defects in the development of type 2 diabetes (DeFronzo and Tripathy, 2009). Based on the expression of myosin heavy chain (MHC) isoforms, mitochondrial content and metabolic properties, skeletal muscle fibers have been classified as either fast-twitch fibers (type IIa, IIb, and IIx) or slow-twitch fibers (type I) (Berchtold et al., 2000). Type IIb and IIx fibers generate ATP mainly through glycolytic pathways, while type I and type IIa contain high concentrations of mitochondria and rely predominantly on oxidative metabolism. Previous studies have demonstrated that glycolytic capacity is increased while oxidative capacity is decreased in skeletal muscle in patients with type 2 diabetes (Simoneau and Kelley, 1997; Gaster et al., 2001; He et al., 2001; Mogensen et al., 2007). Impaired fuel oxidation and mitochondrial function have been proposed to play a causal role in skeletal muscle insulin resistance (Mootha et al., 2003; Mootha et al., 2004; Sparks et al., 2005). That is why so far most of the studies in skeletal muscle have focused on the regulation of oxidative muscle metabolism, leading to the identification of a number of key regulators for oxidative muscle metabolism, such as PGC-1α, AMPK, and SIRT1. Paradoxically, muscle-specific PGC-1α transgenic mice have increased mitochondrial content and slow-twitch myofibers, yet are not protected from high fat diet (HFD)-induced insulin resistance (Lin et al., 2002; Calvo et al., 2008). Notably, recent studies indicated that enhanced glycolytic capacity in individual myofiber strongly correlates with whole body insulin sensitivity (Nader and Esser, 2001; Oberbach et al., 2006; Izumiya et al., 2008), suggesting that activation of the glycolytic myofiber program may represent a compensatory mechanism in response to increasing glucose level in type 2 diabetes. However, very little is known about the regulatory mechanisms that control the metabolic properties of glycolytic muscle fibers.

We recently identified BAF60c as a core component of a novel regulatory pathway that drives glycolytic metabolism in skeletal myofibers (Meng et al., 2013). BAF60c is preferentially expressed in fast/glycolytic muscle. We further demonstrated that BAF60c promotes the metabolic and contractile specification of fast-twitch muscle fibers, and is required for maintaining glycolytic metabolism in adult skeletal muscle in mice. At the mechanistic level, BAF60c forms a transcriptional complex with Six4 to stimulate the expression of Deptor, leading to Deptor-dependent AKT activation in a cell-autonomous manner. To our surprise, oxidative to glycolytic shift in skeletal muscle by BAF60c transgenic expression improves whole body glucose homeostasis in diet-induced obese mice. Our studies identified a new pathway that drives the specification of glycolytic muscle metabolism and provided evidence suggesting that enhanced glycolytic metabolism is a compensatory response to elevated blood glucose levels in type 2 diabetes, and that inadequate adaptation of glycolytic metabolism may accelerate the progression of this disorder. Consistently, we found that expression of BAF60c and Deptor was downregulated in skeletal muscle from severe diet-induced and genetically obese mice (Meng et al., 2013; Meng et al., 2014).

We next went on to dissect whether dysregulation of BAF60c-Deptor-AKT pathway in skeletal muscle contributes to the pathogenesis of type 2 diabetes and what are the upstream signalling cascades that are responsible for this dysregulation. Chronic low-grade inflammation, or meta-inflammation, has been implicated as a key risk factor for obesity-associated insulin resistance (Gregor and Hotamisligil, 2011; Osborn and Olefsky, 2012). However, the underlying mechanisms linking meta-inflammation to skeletal muscle insulin resistance and glucose intolerance remain unclear. We hypothesize that BAF60c-Deptor-AKT pathway may serve as a link between meta-inflammation and skeletal muscle insulin resistance. Indeed, we found that expression of BAF60c and Deptor is downregulated by proinflammatory cytokines, such as TNFα (Meng et al., 2014). Activation of ERK signalling cascade rather than other mitogen-activated protein kinases (MAPKs) or NF-κB pathways is responsible for this dysregulation. Importantly, transgenic rescue of this pathway by muscle specific BAF60c transgenic attenuates muscle insulin resistance and improves systemic glucose homeostasis in both HFD-induced or genetically obese mice. These studies identified BAF60c-Deptor-AKT pathway as a molecular link between meta-inflammation in skeletal muscle and systemic glucose homeostasis.

### BAF60c serves as a glucose sensor in skeletal muscle

Having established the important roles of BAF60c-Deptor-AKT pathway in the specification of glycolytic muscle metabolism and in linking meta-inflammation in skeletal muscle to systemic glucose homeostasis, we went on to explore the role of this pathway under physiological conditions. Interestingly, we recently found that BAF60c functions as a glucose sensor in skeletal muscle and is important for the maintenance of postprandial glucose homeostasis (Meng et al., 2017). In cultured skeletal myocytes, glucose, but not other nutrients, such as fatty acids and amino acids, induces BAF60c and Deptor expression and stimulates AKT activation in a dose-dependent manner. Further mechanistic studies revealed that glucose triggers an ATP sensitive potassium channel (K_ATP_)-dependent Ca^2+^ response, which elicits the phosphorylation and nuclear exclusion of HDAC5 downstream of CaMKII activation, resulting in BAF60c and Deptor induction, and finally leading to insulin-independent AKT activation. This glucose sensing pathway works in concert with the insulin signaling pathway to maintain postprandial glucose homeostasis. Transgenic activation of this pathway in mice lowers postprandial blood glucose levels and improves glucose homeostasis. In contrast, inactivation of this pathway by muscle specific BAF60c deletion exhibits higher postprandial blood glucose levels and exacerbates obesity associated insulin resistance and glucose intolerance. In addition, we discovered that the BAF60c-mediated glucose sensing pathway functions as an extra-pancreatic target of sulfonylurea drugs to exert their full glucose lowering effects.

### BAF60c in hepatic lipid metabolism

Hepatic transcriptional factors that play crucial roles in the regulation of lipogenic gene expression include the upstream stimulating factor (USF), sterol regulatory element-binding protein-1c (SREBP-1c), liver X receptor (LXR), and carbohydrate-responsive element-binding protein (ChREBP). SREBP-1c and USF belong to the helix-loop-helix-leucine zipper family. SREBP-1c binds as dimers to sterol regulatory elements (SREs) on the promoters of lipogenic genes (Yokoyama et al., 1993). USF acts as a molecular platform to recruit distinct transcription factors and cofactors in response to fasting/refeeding via its ability to strongly bind to E-box regulatory elements (Latasa et al., 2003). In response to insulin treatment, USF bound to the E-box recruits SREBP-1c to the nearby SREs. In a recent study, Wang et al. reported that BAF60c physically interacts with USF-1, and is important for the induction of lipogenic genes in response to insulin and feeding (Wang et al., 2013). Upon insulin treatment, USF-1 is phosphorylated by DNA-dependent protein kinase (DNA-PK) and acetylated by P300/CBP-associated factor (P/CAF). In the meantime, BAF60c is also phosphorylated, leading to its translocation from cytosol to nucleus. Direct interaction of phosphorylated BAF60c with phosphorylated/acetylated USF-1 allows the recruitment of SWI/SNF chromatin remodeling complex to activate the transcription of target genes involved in lipogenesis. These results indicate an important role of hepatic BAF60c in insulin-induced lipogenesis.

## BAF60B IN METABOLIC CONTROL AND DISEASES

BAF60b, encoded by *Smarcd2* gene, is the third member of the BAF60 family and is highly expressed in immune cells instead of metabolic tissues. To date, very little is known about its role in metabolic regulation. Recent studies identified BAF60b as a critical regulator of granulocyte development and function (Michel and Kadoch, 2017; Priam et al., 2017; Witzel et al., 2017). Given the fact that metabolic disease is associated with chronic low-grade meta-inflammation, it is possible that BAF60b may indirectly regulate energy metabolism and the development of metabolic syndrome through modulating the immune system and meta-inflammation.

## CONCLUSION

Taken together, accumulating evidence strongly suggests that the SWI/SNF chromatin-remodeling factors BAF60s are important regulators of energy metabolism and their dysregulation plays critical roles in the pathogenesis of metabolic syndrome. However, the current knowledge is limited to the roles of BAF60a and BAF60c in liver and skeletal muscle. Besides these two tissues, other tissues such as pancreas, adipose tissue, and brain are also critical for the development of metabolic syndrome. In addition, BAF60b may exert significant impacts on whole body energy homeostasis by regulating the development and function of the immune system and meta-inflammation. Further studies are needed to address these questions. A better understanding of the exact roles and the underlying mechanisms of BAF60 proteins in metabolic control may provide new insights into the pathogenesis of metabolic disorders such as type 2 diabetes and its associated complications, and form the basis for new therapeutic interventions for these diseases.
